# A new statistical method to analyze Morris Water Maze data using Dirichlet distribution

**DOI:** 10.12688/f1000research.20072.2

**Published:** 2019-10-28

**Authors:** Marianne Maugard, Cyrille Doux, Gilles Bonvento

**Affiliations:** 1 Commissariat à l’Energie Atomique et aux Energies Alternatives, Département de la Recherche Fondamentale, Institut de Biologie François Jacob, Fontenay-aux-Roses, 92260, France; 2 Neurodegenerative Disease Laboratory, Centre National de la Recherche Scientifique, Université Paris-Sud, Université Paris-Saclay, Fontenay-aux-Roses, 92260, France; 3 AstroParticule et Cosmologie, Université Paris Diderot, Paris, 75205, France; 4 Department of Physics and Astronomy, University of Pennsylvania, Philadelphia, PA, 19104, USA

**Keywords:** Morris Water Maze, Statistical analysis, Dirichlet distribution

## Abstract

The Morris Water Maze (MWM) is a behavioral test widely used in the field of neuroscience to evaluate spatial learning memory of rodents. However, the interpretation of results is often impaired by the common use of statistical tests based on independence and normal distributions that do not reflect basic properties of the test data, such as the constant-sum constraint. In this work, we propose to analyze MWM data with the Dirichlet distribution, which describes constant-sum data with minimal hypotheses, and we introduce a statistical test based on uniformity (equal amount of time spent in each quadrant of the maze) that evaluates memory impairments. We demonstrate that this test better represents MWM data and show its efficiency on simulated as well as *in vivo* data. Based on Dirichlet distribution, we also propose a new way to plot MWM data, showing mean values and inter-individual variability at the same time, on an easily interpretable chart. Finally, we conclude with a perspective on using Bayesian analysis for MWM data.

## 1 Introduction

The Morris Water Maze (MWM) was first described by Richard Morris in the 80’s 1 and is still one of the most commonly used tasks to evaluate spatial learning in rodents, including normal and genetically modified mice. While the standard reference memory task is mostly used and is validated as an assay for hippocampus-dependent spatial navigation and reference memory, modifications of the basic protocol allow to also evaluate reversal learning, the delayed match to place task and procedures for dissociating encoding and retrieval. At least for the standard reference memory and reversal learning tasks, these procedures require probe test data that display a constant-sum constraint. The maze consists of a large circular tank filled with opaque water in which rodents can escape onto a platform hidden just beneath the surface. During a training phase animals perform repeated blocks of 60 second-long trials to find the location of a fixed platform using distant visual cues from semi-random start locations and the escape latency is recorded. Since data are right-truncated at 60 seconds, in contradiction with a normal distribution and causing potentially biased results, statistical guidelines have been published to properly characterize learning behaviors using survival data 2.

During a probe test session, the platform is removed and animals freely navigate into the pool from the same start location and for the same fixed amount of time ( *e.g.* 60 seconds). The path of the animal is recorded using a video camera and an automatic tracking system. Data collected during the probe test session can be classified into three categories: time spent per zone, which can be theoretical quadrants defined on the pool or a theoretical annulus drawn around the platform location; number of crossings of the platform area; or total proximity to the platform center 3. Creating a large database using several published tests from their institute and simulated data, Maei et al. have shown that total proximity allows the best detection for small samples, whereas time spent in quadrants is of great interest for bad performers 4. Since this test is often used to characterize memory loss, time spent in theoretical quadrants is mostly found in the literature.

Several hypotheses can be tested using data obtained from time spent in quadrants: ’Can one group of rodent remember the platform location?’ or ’Is there any difference of memory abilities between several groups of rodents?’. In both cases, the statistical analysis of the data often focuses on the target quadrant ( *e.g*. that where the platform was placed during the learning phase) using parametric tests like ANOVAs and *t*-tests ^1^. These tests are based on normal distributions and independence, which cannot be accurately assumed in this context since 1) variables are defined on a finite interval and 2) variables corresponding to the time spent in the four quadrants are necessarily anti-correlated. Moreover, these tests neglect the time spent in the three other quadrants inducing a loss of information and hiding the aspect of preference for one quadrant that is supposed to reflect efficient spatial memory. Some authors used non-parametric alternatives, but even if their use may be preferable with the sample size of behavioral studies, they still do not fully describe the experiment. These observations suggest that a better characterization and a more suitable statistical analysis of data obtained through the MWM could significantly improve the accuracy of the results.

**Figure 1.**
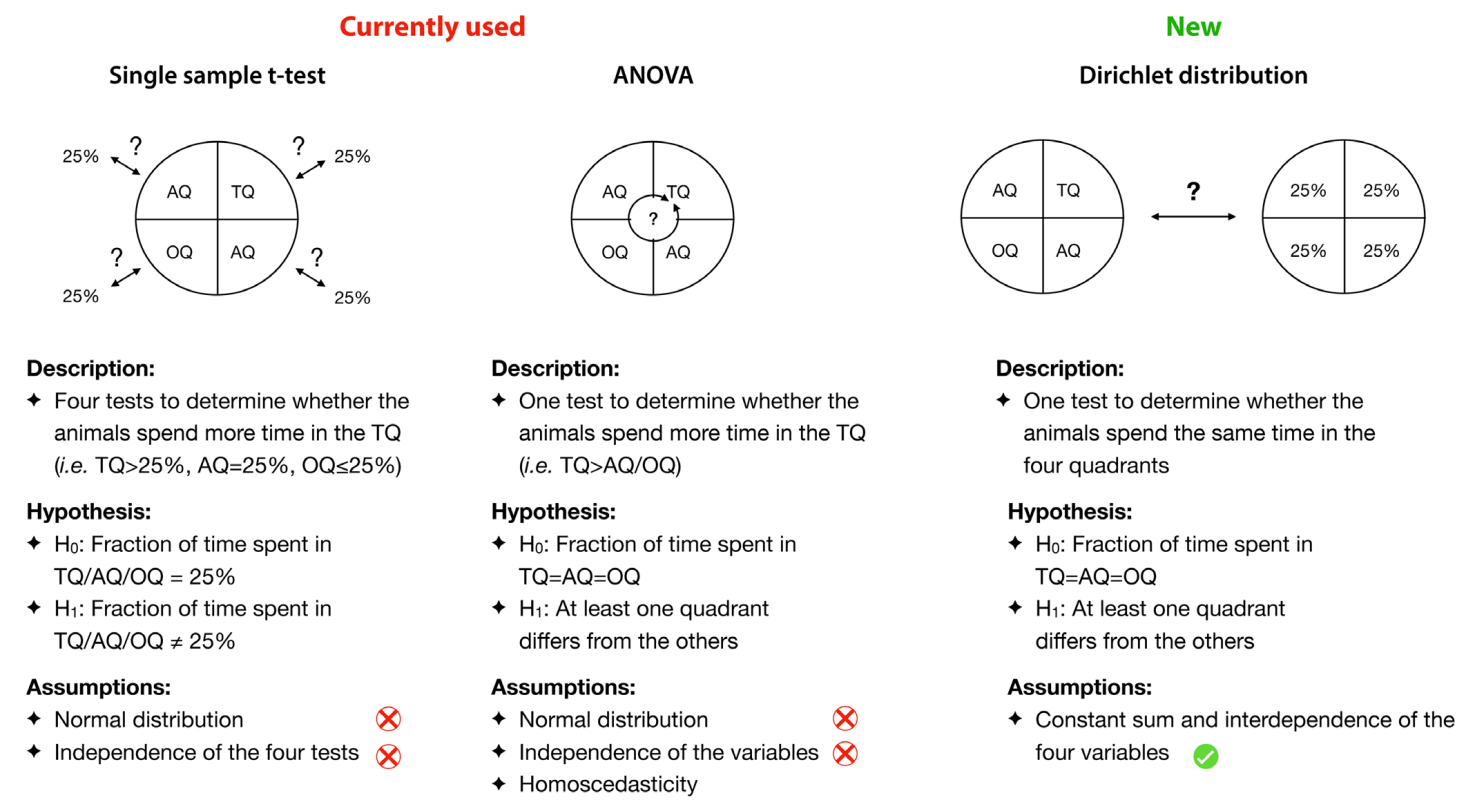
Summary of the analyses of the MWM that are currently used in comparison with Dirichlet distribution. To determine whether one group of animals has a preference for a quadrant one usually uses *t*-tests or ANOVAs assuming normal distribution and independence. Those assumptions do not describe correctly the dataset obtained from a MWM. In comparison, the Dirichlet distribution allows to answer the same question but describes properly the constant sum constraints and interedependence of the variables.

Focusing on the question ’Can one group of rodents remember the platform location?’ to evaluate memory abilities, we suggest to use the Dirichlet distribution, a distribution that describes several variables with a constant sum, to collectively describe the fraction of time spent in the four quadrants of the maze. This test would provide a unique *p*-value allowing determination of whether the rodents spent the same amount of time in the four quadrants or not, a primary indication of significant spatial memory. In the case of differences between the four quadrants, this test can be followed by four *post-hoc* Student *t*-tests to identify preference or aversion for some quadrants. In comparison, the currently used method ( *i.e*. directly using the Student *t*-test on the target quadrant) does not allow to identify memory loss and may hide some bias ( Figure 1).

We will first describe the methodology we developed with the Dirichlet distribution and the correction required to fit with the sample size of behavioral experiments. Using simulated data we will show that beyond the better description of the results, using Dirichlet distribution allows reducing the number of false positives and false negatives, significantly improving the reliability of the analysis. We then applied this test on *in vivo* data to validate its use in experimental conditions, also providing a way to graphically present the data that takes into account interindividual variability. Finally, we will discuss the advantages and limits of the application of the Dirichlet distribution on behavioral studies, broaching the major inputs that using Bayesian inferences could bring in this field of research. 

## 2 Methods

### 2.1 The Dirichlet distribution

The Dirichlet distribution is a multivariate generalization of beta distributions. It describes the distribution of *K*-dimensional vectors p for which the sum of all the coordinates is fixed, *i.e.* $\sum_{k=1}^{K}p_{k}=1$. It is parametrized by a *K*-dimensional vector ***α*** of positive reals *α _k_ >* 0, 1 ≤ *k* ≤ *K*, such that its probability density function is given by

$$(p|\;\alpha )=\frac{1}{B(\alpha )}\prod_{k=1}^{K}p_{k}^{\alpha_{k}-1},\;(1)$$

where $B(\alpha )=\prod_{k=1}^{K}\text{Γ}(\alpha_{k})/\text{Γ}(s)$ is the multivariate beta function and $s=\sum_{k=1}^{K}\alpha_{k}$ is the *precision*. The marginal distributions are beta distributions with parameters ( *α _k_*, *s* – *α _k_*) with expectation values *m _k_* = *α _k_*/ *s*, variance *m _k_* (1 – *m _k_*)/( *s* + 1) and covariance between coordinates *p _i_* and *p _j_* given by – *m _i_m _j_*/( *s* + 1). Therefore, the higher the precision *s*, the less diffuse coordinates are around their means. The Dirichlet distribution is the most general distribution for fixed-sum variables, motivating its use to describe compositional or fractional data such as MWM data, where *K* = 4. The likelihood of a sample of *N* independent observations D = {p _1_, . . . , p _N_} is given by

$$(D|\alpha )=\prod_{i=1}^{N}(p_{i}|\alpha ).\;(2)$$

### 2.2 Likelihood-ratio test based on the Dirichlet distribution

**Description of the test** To reflect memory abilities, we would like to test whether the fraction of time spent in the four quadrants significantly differs from a uniform distribution, thus showing preference for one or several quadrants. To do so, we propose a likelihood-ratio test based on the Dirichlet distribution to distinguish between the null hypothesis of uniformity *H*_0_ : {∃ *α >* 0, ∀ *k*, *α _k_* = *α*} (implying that all means *m _k_* are equal to 1 */K* but the precision is not constrained), and the general hypothesis *H*_1_ where the *α _k_*’s are unconstrained. The likelihood-ratio statistic reads

$$\Lambda =2\times \left[\underset{\alpha \in H_{1}}{\sup\;}\ln\;(D|\alpha )-\underset{\alpha \in H_{0}}{\sup\;}\ln\;(D|\alpha )].\;(3\right)$$

In order to fit the distribution parameters to their maximum likelihood values, we refer to the numerical schemes developed in 5 and we used the open-source Python module dirichlet implemented by Eric Suh ^2^ and run with Python 3.6. In particular 5, proposes a technique to alternatively fit the means *m _k_* and precision *s*, faster than fitting directly the *α _k_*’s. The maximum likelihood parameters under the null hypothesis are thus estimated by setting the means to their uniform value, *m _k_* = 1 */K*, and fitting the precision *s*. We provide a slightly modified version of the dirichlet package, forked from that of Eric Suh, which is publicly available ^3^. Under the null hypothesis, the likelihood-ratio statistic Λ asymptotically follows a *χ* ^2^-distribution with *K* – 1 degrees of freedom.

**Figure 2.**
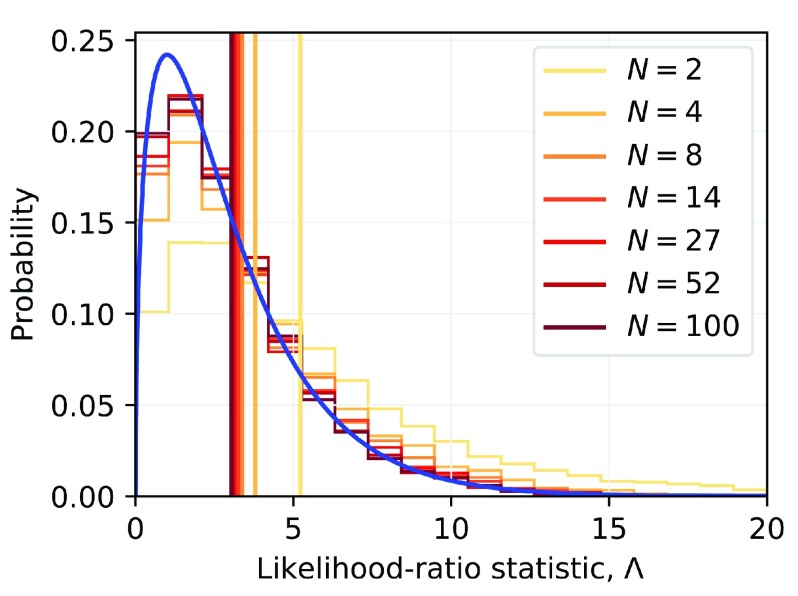
Histogram of the likelihood-ratio statistic Λ for *s* = 10 for various sample sizes *N*. Histograms of the likelihood-ratio statistic Λ are represented in different colors according to the N value. Means are represented by vertical lines of the same color. The $\chi_{3}^{2}$-distribution is represented in blue. For small samples, the distribution of the likelihood ratio slightly deviates from a $\chi_{3}^{2}$ distribution and the mean is significantly greater than the theoretical value of 3.

**Bartlett correction** Biological samples are usually limited and for small samples the statistic’s distribution deviates from a $\chi_{K-1}^{2},$ as can be seen in Figure 2. We propose an approximate Bartlett-type correction 6 for small samples, which amounts to rescale the likelihood-ratio statistic to match its asymptotic mean, which is *K* – 1 in this test. Such a correction has been shown to correctly reproduce the first three moments of the asymptotic *χ* ^2^-distribution 6. In order to derive the scaling factor, we needed to compute the expected value of the statistic as a function of the number of samples *N* and the precision *s*. To do so, we drew random samples of *N* observations from uniform Dirichlet distributions with precision *s*, varying *N* between 2 and 100 and *s* between 1 and 1000 (with logarithmically-spaced values), and measured the mean of the statistic Λ ^4^. We found that the mean value of the likelihood-ratio statistic Λ depends very little on *s* in the probed range, and that the difference to the asymptotic value of *K* – 1 is well-fitted with a power law in *N*, *i.e.* 〈Λ〉 – ( *K* – 1) ~ *a _K_*N*^b _K_^* (data not shown). We found the approximate values *a _K_* = 5.9 and *b _K_* = –1.4 for *K* = 4. We therefore propose to use a corrected statistic

$$\tilde{\text{Λ}}\equiv \text{Λ}\times \frac{\left(K-1\right)}{(K-1)+a_{K}N^{b_{K}}},\;(4)$$

and to compare its observed value to the *χ* ^2^ expected value corresponding to the desired statistical significance.

**Figure 3.**
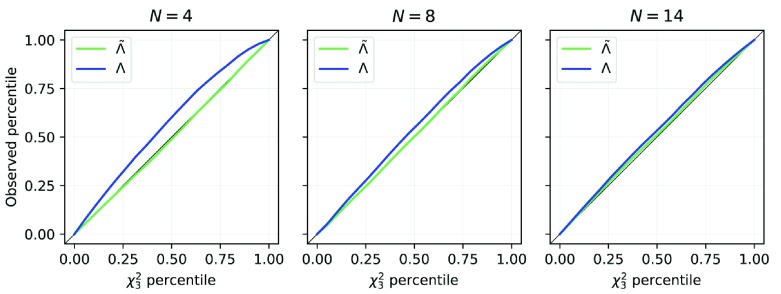
Probability-probability plots for uncorrected and corrected statistics from our simulated data. The uncorrected and corrected statistics are compared with a $\chi_{K-1}^{2}$ distribution for several sample sizes *N*. Grey lines represent equality between Λ percentiles and $\chi_{K-1}^{2}$ percentiles. Blue lines correspond to the uncorrected statistics and green lines to the corrected one. There is a difference between the *p-*values from the uncorrected statistics and the theoretical ones that disappears after correction, especially for small sample size.

**Validation of the test** To validate this correction, we compared the distribution of the uncorrected statistic Λ and the corrected statistic $\tilde{\Lambda }$ from our simulated samples to a $\chi_{K-1}^{2}$-distribution with probability-probability plots. As shown in Figure 3, the uncorrected statistic yields *p*-values significantly different from the theoretical ones while the corrected *p*-values are in perfect agreement with the $\chi_{3}^{2}$-distribution. Therefore, this correction significantly improves the reliability of the test for small samples.

**Figure 4.**
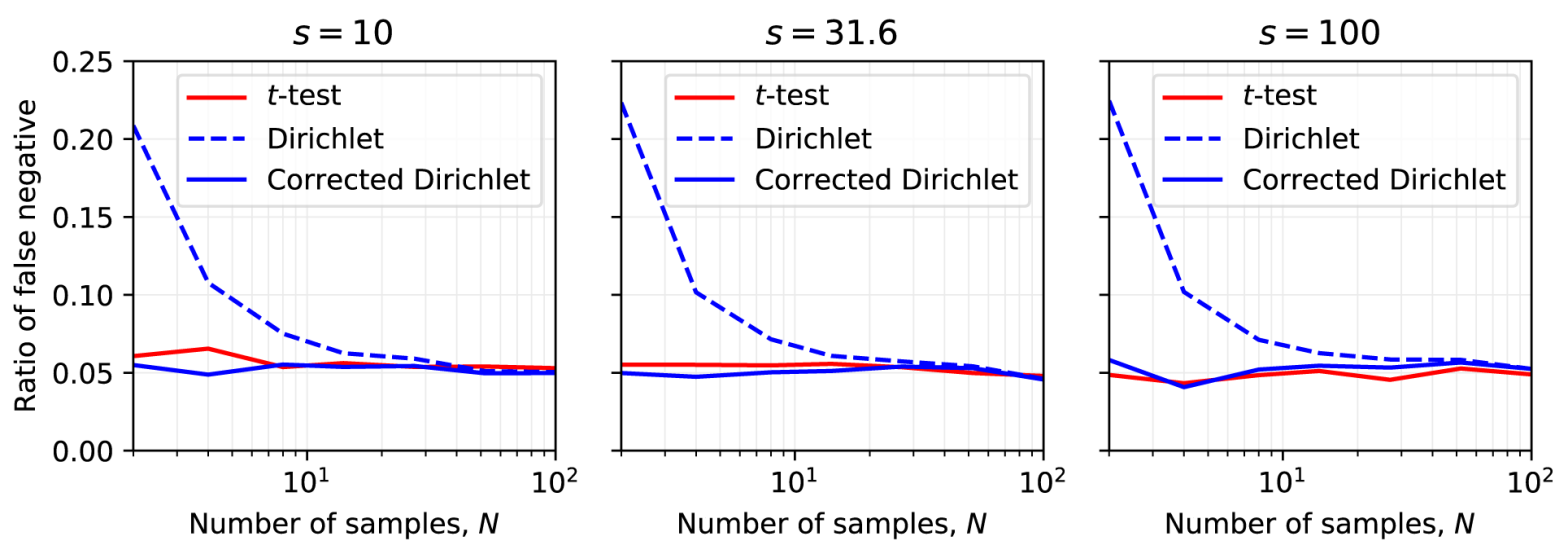
Rate of false negatives depending on the sample size *N* for different values of the precision *s* for a *p*-value of 0.05. The rate of false-negative ( *i.e. * type 1 error) using the corrected version of the statistic is represented by the blue line whereas the rate of false-negative using the uncorrected version of the statistic is represented by the blue dotted line. We found that the correction results in a consistent rate of false negatives, independent of the sample size *N* and precision *s* in the range tested. The red line represents the rate of false-negative using a single-sample *t*-test on the target quadrant.

We also computed the number of false negatives on the simulated data to evaluate the rate of type 1 error ( Figure 4). We found that using the *p*-value from the corrected statistic leads to a consistent rate of type 1 error ( *i.e. α* =5%), independent on the number of samples *N* or the precision s. On the contrary, using the non-corrected statistic leads to more false negatives, especially when the number of samples is small.

**Comparison with the one-sample Student *t*-test** In order to compare the type 2 error obtained with the Dirichlet distribution with the results obtained using a one-sample *t*-test on the target quadrant as often done in the literature, we simulated data from a non-uniform Dirichlet distribution with the parameters ***α*** = (40; 20; 20; 20). We found that the *p*-value from the corrected statistic of the Dirichlet distribution is mainly lower than the one obtained with a single *t*-test on the target quadrant (75% of the *p*-values are lower for *s* = 30). This means that for some cases where the target quadrant is preferred, using a one-sample *t*-test on the target quadrant would not detect this preference whereas the test based on Dirichlet distribution would detect the divergence from uniformity. Beyond improving the description and the interpretation of data from the MWM, the test we propose extracts more information from the same experiment as it is based on a larger dataset and then decreases the number of false-positives.

**Post-hoc analysis** Using the test based on Dirichlet distribution, we can determine whether the fraction of time spent in the quadrants is uniformly distributed. In the case of a divergence from uniformity, we would like to evaluate what are the quadrants responsible for this divergence as a *post-hoc* analysis.

This can be performed by comparing the marginal distributions of each quadrant, that are Beta distributions, to a theoretical Beta distribution with parameters *α* = 0.25 *s* and *β* = 0.75 *s*. The only simple way to compare one distribution with a theoretical one is to apply a single sample t-test that compares a normal distribution with a theoretical normal distribution. However, we noticed that in the range of inter-individual variability we have in this kind of study (given by the parameter *s* of the Dirichlet distribution, usually found between 20 and 50), the marginal distribution are fairly close to a normal distribution. Seeking for simplification, we advise to apply single sample *t*-tests for a *post-hoc* characterization of the preference for a quadrant in groups showing a divergence from uniformity.

### 2.3 Bayesian inference of the parameters of the Dirichlet distribution

Bayesian analysis can be used to infer constraints on the parameters *α _i_*’s of the Dirichlet distribution used to model the data (and subsequently the means *m _i_*’s). Specifically, we performed nested sampling of the parameter space of the Dirichlet distribution using the PyMC3 package with Python 3.6. For simplicity, we used the Jeffreys prior ^5^ *π*( ***α***) which does not depend on the model parametrization ( *e.g.*, sampling over ***α*** or ( **m**, *s*)) and leave the discussion about this choice for future work. The output is a sample of vectors ***α*** distributed as the posterior given the data, *i.e.* ( ***α*** | **D**) ∝ ( **D** | ***α***) *π*( ***α***), which enables us to compare confidence regions for different groups and visualize the consistency with uniformity.

## 3 Results

We used a dataset obtained comparing memory abilities of female *wild-type* mice to female 3xTg AD mice, a model for Alzheimer’s Disease 7. All information related to experimental and ethical procedures are available in 8.

### 3.1 Application of the likelihood-ratio test based on the Dirichlet distribution

**Figure 5.**
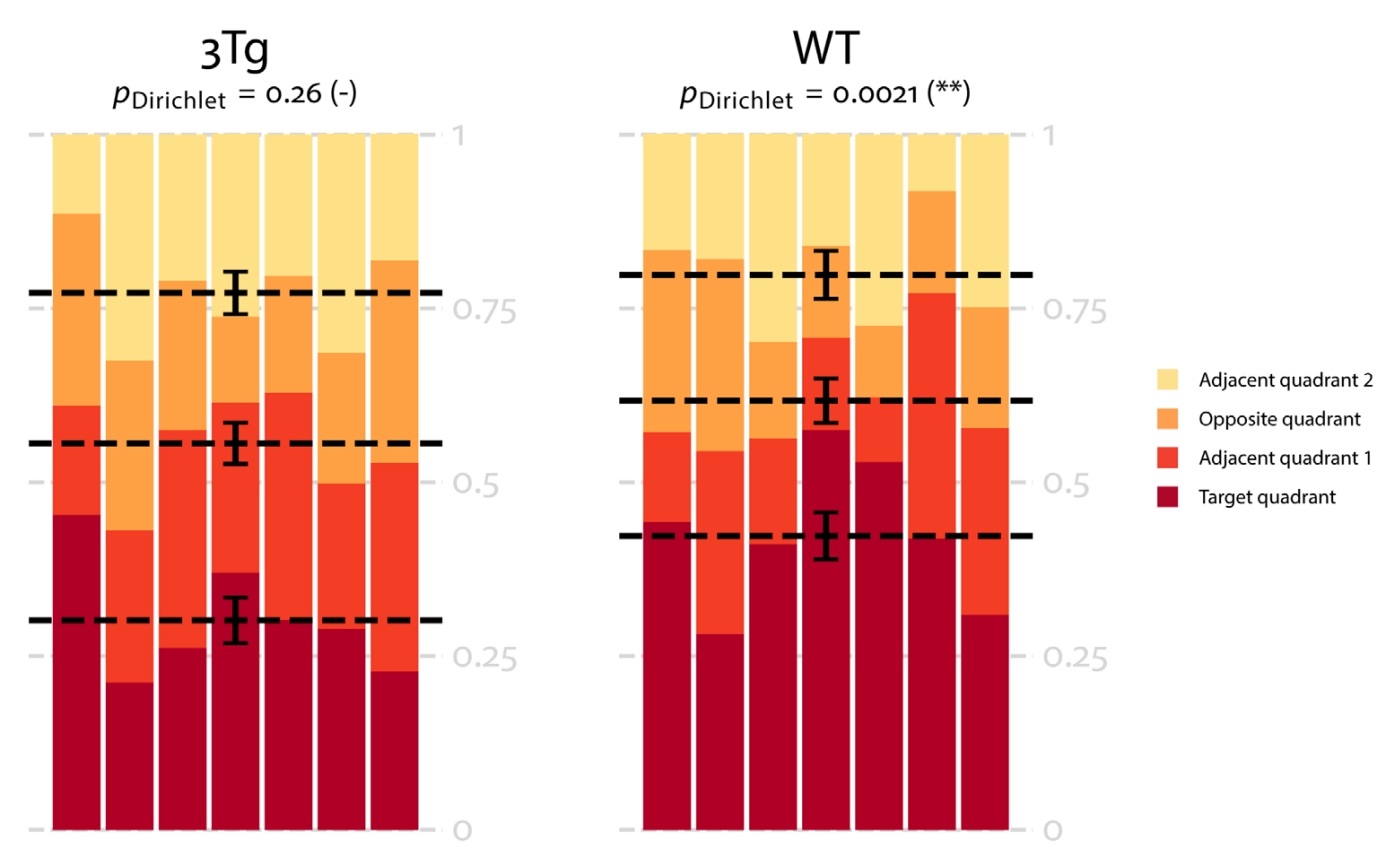
Time spent in the four quadrants by *wild-type* and 3xTg AD mice. In this plot, each column represents a sample and each color represents a quadrant. Mean values for the fraction of time spent in each quadrant is represented by a dotted line and the error bars on the means are approximated with the inverse Fisher information. For 3xTg mice the fraction of time spent in each quadrant is approximately similar leading to a uniform distribution ( *p* = 0.26) whereas for *wild-type* mice the time spent in the target quadrant is significantly higher leading to a non-uniform distribution ( *p* = 0.0021).

We compared the distribution obtained for each group to a uniform distribution in the probe test of the standard reference memory task and we found that the Dirichlet distribution obtained for *wild-type* mice was significantly different from a uniform distribution ( *p* = 0.0021), whereas the one obtained for 3xTg mice did not differ from a uniform distribution ( *p* = 0.26). We also propose a module, included in the Dirichlet package, to draw charts showing at the same time mean values with uncertainties ^6^ and inter-individual variability according to Dirichlet distribution ( Figure 5). This result shows that 3xTg AD mice display long term memory deficits, which is in accordance with previous observations 9.

To better characterize long term memory in *wild-type* mice, we applied single sample *t*-tests on the four quadrants as a *post-hoc* analysis. We performed a one-tailed single sample *t*-test to assess whether the fraction of time spent in the target quadrant by *wild-type* mice is greater than the theoretical value 25%. Conversely, we performed a one-tailed single sample *t*-test to assess whether the fraction of time spent in the opposite quadrant by *wildtype* mice is lower than the theoretical value 25%. For adjacent quadrants we performed two-tailed single sample *t*-tests. We observed that the fraction of time spent in the target and opposite quadrants were respectively significantly higher ( *p* = 0.025) and lower ( *p* = 0.013) than 25%. The fraction of time spent in the adjacent quadrants did not differ from 25%.

Using this dataset with usual sample sizes for behavioral studies (N=7), we confirmed that our test is able to discriminate efficient and deficient memory abilities on real data.

### 3.2 Perspectives using Bayesian inference

**Figure 6.**
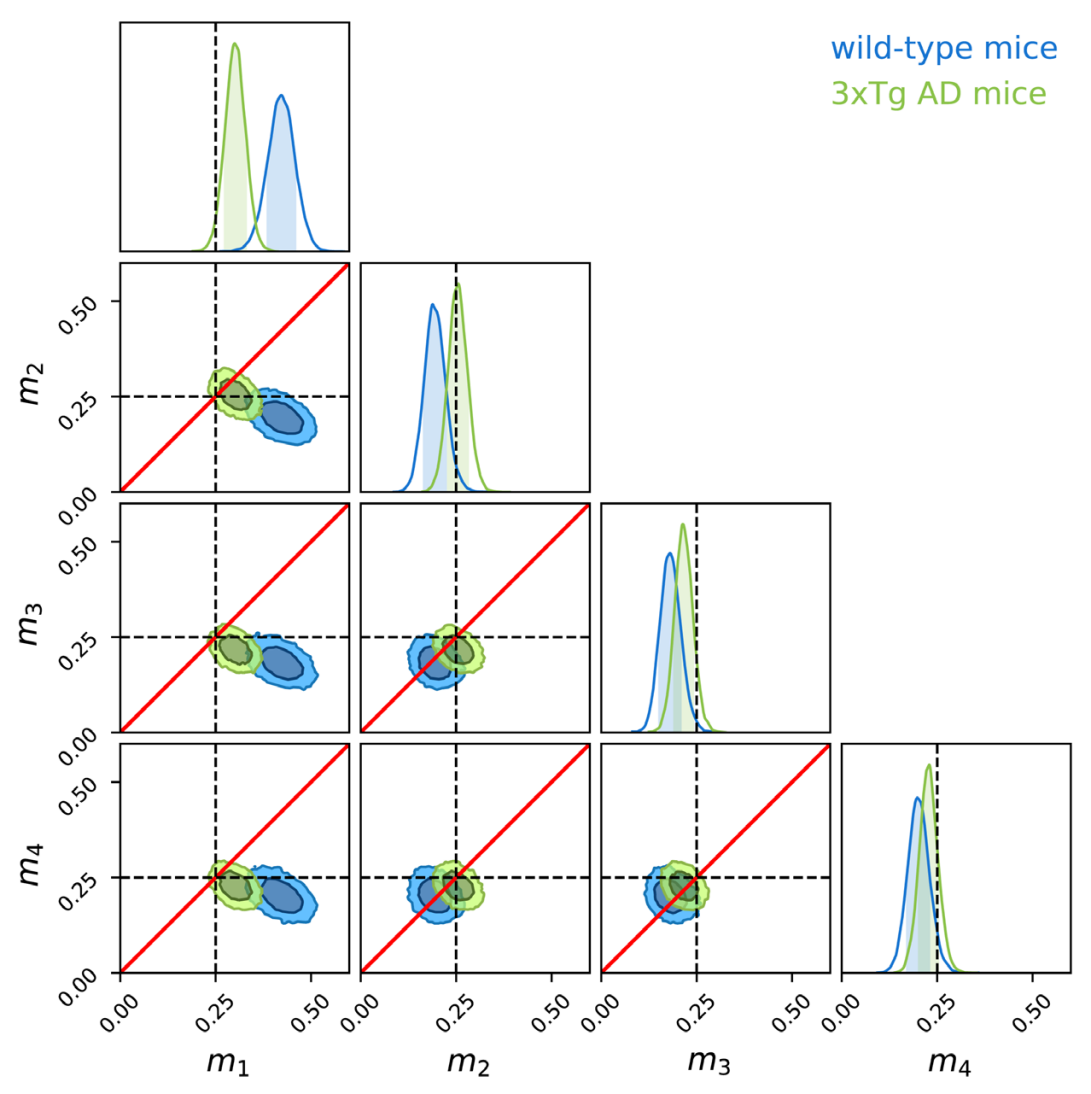
Fraction of time spent in the four quadrants for *wild-type* and 3xTg AD mice in the case of Bayesian inference. Corner plot representing constraints on the mean fractions of time *m _i_*’s for the two data sets *wild-type* (blue) and 3xTg (green). The diagonal plots show the marginal distributions of *m _i_*’s (with shaded 68% confidence interval) and off-diagonal plots show the two-dimensional distributions of pairs of these variables (inner and outer contours represent the 68% and 95% confidence levels). The black dashed lines represent the case of uniformity (25%) and the red lines correspond to equal time spent in both considered quadrant. Constraints on *m*_1_ (leftmost column) indicate that *wild-type* mice favor the target quadrant.

We inferred constraints on the parameters of the Dirichlet distribution for *wild-type* and 3xTg mice. Figure 6 indicates compatibility of the data with uniformity for 3xTg mice and shows a clear preference for the target quadrant for *wild-type* mice suggesting memory deficits in 3xTg mice compared with *wild-type*.

## Discussion

We proposed a statistical approach for the analysis of MWM probe test data based on the Dirichlet distribution as a model for the fraction of time spent by rodents in the quadrants of the maze. In the context of behavioral experiments that usually generate a lot of data with high inter-individual variability, a lot of parameters can be taken into account to extract evidence of memory abilities 3. In the literature, the time spent in quadrants – the target quadrant, but sometimes also the opposite quadrant 9, 10 – is commonly used to assess long-term memory. Even if the focus on the time spent in quadrants is broadly accepted as a good index to evaluate reference memory, there is no consensus about the processing of these data. In this context, the Dirichlet distribution has the great advantage to simultaneously take into account the four quadrants and to correctly account for the constant-sum constraint of such data, which implies both deviation from the normal distribution and interdependence. That way, it gives a correct description of the data obtained from MWM probe tests and provides meaningful plots representing mean performances and inter-individual variability.

We showed that the corrected test based on the Dirichlet distribution gives a consistent rate of false-negative, even for small sample size. This indicates that this test can be safely used even in the context of behavioral studies with sample size smaller than 10 individuals, as we confirmed using the results previously obtained on *wild-type* and 3xTg AD mice.

Beyond the great improvement in the description of MWM probe test data, we also showed that this test gives less false-negatives than its inaccurate but commonly used alternative, the Student *t*-test. Therefore, using Dirichlet distribution is the best option to extract reliable information from time spent in quadrants during a MWM probe test. Combination of this analysis with results based on other measures of performance will give a comprehensive and accurate description of rodent memory abilities.

However, there are two main limitations in the use of the likelihood-ratio test based on the Dirichlet distribution: 1) it cannot directly identify the preferred quadrant and 2) it cannot compare memory abilities between several groups of animals. We proposed to overtake the first limitation by performing a *post-hoc* analysis to determine which quadrants are responsible for divergence from uniformity. We showed that performing single-sample *t*-tests, the only existing statistical test comparing one distribution with a theoretical one, as a *post-hoc* analysis (instead of *ad-hoc*) is satisfying. However, more interesting results can be obtained using Bayesian statistics, a method that can also permit comparison between groups. Deriving informative *p*-values on binary tests from such analysis remains challenging but represents an active field of research that could soon provide a great opportunity to improve MWM statistical analyses.

## Conclusion

We propose here a new way to analyze MWM probe test data from the standard reference memory task of the MWM that accurately and simultaneously describes the four variables of time spent in the quadrants and allows to extract more information from the same experiments than the currently used method. All the packages required to perform the statistical test and to draw the corresponding chart are publicly available ^7^ and can be easily run with R using the reticulate package 11. Minor modifications of this test would allow to apply the same methodology on other behavioural tests facing the same constraints like H-Maze or Y-Maze.

## Data availability

### Underlying data

A Python notebook with the code to reproduce the simulations and figures is available at https://github.com/xuod/dirichlet 12. *In vivo* dataset available from: https://github.com/xuod/dirichlet/tree/master/example 12. (Experimental procedures and data acquisition are detailed in 8).

## Software availability

Dirichlet package from Eric Suh (Fitting the parameters of a Dirichlet distribution) available from: https://github.com/ericsuh/dirichlet

License: MIT

Dirichlet package used in the present study (Likelihood-ratio test based on Dirichlet distribution) available from: https://github.com/xuod/dirichlet/tree/master/dirichlet

Archived package as at time of publication: http://doi.org/10.5281/zenodo.3373955 12

License: MIT

## Footnotes

^1^Among the 30 most recent articles using the Morris Water Maze test and published at the end of October 2018, 25 used time spent in quadrants as a criterion. 23 used a parametric test (Student *t*-test or ANOVA) to analyze their data, whereas only 2 used a non parametric alternative. 24 out of 25 articles presented data as bar charts without presenting inter-individual variability.

^2^ https://github.com/ericsuh/dirichlet

^3^ https://github.com/xuod/dirichlet

^4^For each tuple ( *N, s*), the number of samples is increased until the means is measured with relative error below 0.01.

^5^For the *K*-dimensional Dirichlet distribution, the Fisher information is *I* ( ****α****) = diag(Ψ _1_ ( **α**)) − *Ψ*_1_ ( *s*) *J*_4_, where *J*_4_ is a *K* × *K* matrix of ones, and the Jeffreys prior is $\pi (\alpha )\propto \sqrt{\left|I(\alpha )\right|}.$

^6^We approximate the variance by the inverse Fisher information, which is a lower bound, given by ${\hat{\sigma }}^{2}(\alpha_{i})=1/{\left(\psi_{1}(\alpha_{i})-\psi_{1}(s)\right)}^{n}$ where *ψ*_1_ is the trigamma function. A full bayesian analysis can be performed to obtain those error bars, as suggested in Section 2.3.

^7^ https://github.com/xuod/dirichlet
