# Supplementary material for: A new statistical method to analyze Morris Water Maze data using Dirichlet distribution
Source: F1000Res. 2019 Oct 28;8:1601. Originally published 2019 Sep 6. [Version 2] doi: 10.12688/f1000research.20072.2 (PMC6833991; doi:10.12688/f1000research.20072.2)
Supplement: Peer review history

# Peer review: Reviewer response for version 2

**DOI:** 10.5256/f1000research.23263.r55813

**Published:** 2019-11-05

**Authors:** Eleni Vasilaki, Avgoustinos Vouros, Luca Manneschi

**Affiliations:** 1 University of Sheffield , Sheffield, UK; 2 Department of Computer Science, University of Sheffield, Sheffield, UK

Thank you for sharing with us a revised version of the manuscript. First, we would like to clarify that approval with reservations intended to be approval with minor corrections.

 Further, we are pleased with the way you have addressed the second point we raised, which was to comment on the applicability of your methods on other types of trials.

 However, we feel that the reply to the first point we raised didn’t add to the manuscript, in fact, it might have even deteriorated the flow of the text. The comment was not meant to be interpreted such as “why you do a t-test”, this is very well justified in the original version. Rather we are asking: “Could you comment whether in the case that the data are not following a Gaussian distribution you can still apply your method?” 

 We will not provide any further reviews, but rather leave it to you whether you would like to improve the manuscript based on the feedback or not.

We confirm that we have read this submission and believe that we have an appropriate level of expertise to confirm that it is of an acceptable scientific standard.

# Peer review: Reviewer response for version 1

**DOI:** 10.5256/f1000research.22038.r53542

**Published:** 2019-09-30

**Authors:** Eleni Vasilaki, Avgoustinos Vouros, Luca Manneschi

**Affiliations:** 1 University of Sheffield , Sheffield, UK; 2 Department of Computer Science, University of Sheffield, Sheffield, UK

The authors suggest a new statistical procedure to analyze in more depth a common performance measurement in the Morris Water Maze experimental procedure.

 The manuscript is well-written and all the methods explained in detail. The new proposed method is well-justified and shows much potential in the field of data analytics for behavioural procedures similar to the Morris Water Maze. Code of all the methods that the author describe is open source and publicly available. Instructions on how to run the code are clear.

 I would like to point out some aspects that could have been addressed/discussed more: 

1. For the post-hoc analysis the authors suggest the usage of t-tests on the four quadrants. Have the authors consider, if applicable, alternative tests of hypothesis in case the assumptions of the t-test (e.g. the data are not normally distributed) are not fulfilled?
2. The authors focus on the probe trials but couldn't analysis on other types of trials (e.g. reversal learning) also been benefit from their method? Can the authors comment if their method is also applicable to other scenarios where there are mutually exclusive possibilities?

We confirm that we have read this submission and believe that we have an appropriate level of expertise to confirm that it is of an acceptable scientific standard, however we have significant reservations, as outlined above.

# Response

**Published:** 2019-10-24

**Authors:** Gilles Bonvento

**Affiliations:** CNRS UMR 9199, France

We thank the reviewers for their positive comments regarding our statistical test.

1. For the post-hoc analysis the authors suggest the usage of t-tests on the four quadrants. Have the authors consider, if applicable, alternative tests of hypothesis in case the assumptions of the t-test (e.g. the data are not normally distributed) are not fulfilled?

As far as we know, there is no test available to compare non-normal distribution with a theoretical one.

1. The authors focus on the probe trials but couldn't analysis on other types of trials (e.g. reversal learning) also been benefit from their method? Can the authors comment if their method is also applicable to other scenarios where there are mutually exclusive possibilities?

Our method can be used each time a probe test is performed with the sum spent in the 4 quadrants totalling 100%, such as for reversal learning.

# Peer review: Reviewer response for version 1

**DOI:** 10.5256/f1000research.22038.r53541

**Published:** 2019-09-23

**Authors:** Richard GM Morris

**Affiliations:** 1 Centre for Cognitive and Neural Systems, University of Edinburgh, Edinburgh, EH8 9JZ, UK

Maugard *et al.* identify a major difficulty in analyzing probe test data from the watermaze which typically consists of time (in sec) or proportions of time (%) spent in each of four quadrants of the pool. While it is straightforward to suppose that more time should be spent in the training quadrant, leading some to analyze only that quadrant, a better method would be to use a mathematical approach that recognizes that the time in all 4 quadrants must add to 100% but their distribution is of interest. Some authors have attempted (upon advice) to address the quadrants problem by reducing the numerator degrees of freedom by 1 (e.g. Morris), but this is unlikely to be statistically adequate, and anyway suffers from issues associated with the normality of the data etc. What is proposed here is something called the Dirichlet distribution which explicitly recognizes the summation to 100% problem and provides a mathematical way of looking at more than just the training quadrant.

 This innovation seems valuable, but I add a qualification. This is that the watermaze is, essentially, no more than a pool of water with a hidden platform in which a large variety of different tasks can be run. That most users of the watermaze use only the standard reference memory task does not mean that other variants are not of interest - reversal learning (Hans-Peter Lipp and David Wolfer, Univ Zurich), the delayed match to place task (Morris, e.g. Steele and Morris Hippocampus 1999 ^1^), and procedures for dissociating encoding and retrieval (e.g. Rossato *et al.* Current Biology 2018 ^2^). These other procedures are of analytical interest. However, they may still require probe test data (e.g. Rossato *et al.* 2018 ^2^).

 My recommendation is that the paper be provisionally accepted - it makes a very valuable point - but attention must be paid to the fact there is not just one single task that can be run in a watermaze. There is greater potential.

 To add also, there are a large variety of measures of performance including latency, path-length, directionality, proximity index and all the measures associated with a probe test. Given this, the thrust of the paper is arguably less novel than the authors surmise, albeit this being a very clever solution to an unsolved problem associated with probe tests.

 Recommendation: index subject to small revisions (as advised above).

I confirm that I have read this submission and believe that I have an appropriate level of expertise to confirm that it is of an acceptable scientific standard.

## References

[1] : Delay-dependent impairment of a matching-to-place task with chronic and intrahippocampal infusion of the NMDA-antagonist D-AP5. *Hippocampus*.1999;9(2) : 10.1002/(SICI)1098-1063(1999)9:2<118::AID-HIPO4>3.0.CO;2-8 118-136 10.1002/(SICI)1098-1063(1999)9:2<118::AID-HIPO4>3.0.CO;2-8 10226773

[2] : Silent Learning. *Curr Biol*.2018;28(21) : 10.1016/j.cub.2018.09.012 3508-3515.e5 10.1016/j.cub.2018.09.012 30415706

# Response

**Published:** 2019-10-24

**Authors:** Gilles Bonvento

**Affiliations:** CNRS UMR 9199, France

We would like to thank the reviewer for his positive comments. We appreciate the explicit acknowledgment regarding our valuable test. We have now mentioned in the text that a large variety of different tasks can be run using modifications of the basic protocol.
